# Incidence of peripheral intravenous catheter failure among inpatients: variability between microbiological data and clinical signs and symptoms

**DOI:** 10.1186/s13756-019-0581-8

**Published:** 2019-07-22

**Authors:** Ian Blanco-Mavillard, Miguel Ángel Rodríguez-Calero, Joan de Pedro-Gómez, Gaizka Parra-García, Ismael Fernández-Fernández, Enrique Castro-Sánchez

**Affiliations:** 1 Quality, Teaching and Research Unit, Hospital Manacor, Cra. de Manacor-Alcudia s/n, 07500 Manacor, Spain; 2 Servei de Salut de les Illes Balears, Palma, Spain; 3 Universitat de les Illes Balears, Palma, Spain; 4 Care, Chronicity and Evidence in Health Research Group, Health Institute of Health Sciences, Palma, Spain; 5 Hospital Sant Joan de Deu, Palma, Spain; 6 NIHR Health Protection Research Unit in Healthcare Associated Infection and Antimicrobial Resistance at Imperial College London, London, UK

**Keywords:** Peripheral intravenous catheter, Catheter failure, Adverse events, Catheter-related bloodstream infections, Vascular access device

## Abstract

**Background:**

Peripheral intravenous catheters (PIVCs) are the most widely used invasive devices among inpatients. Catheter-related bloodstream infections (CRBSI) are serious yet preventable events for patients. Although the contribution of PIVCs towards these infections is gradually being recognised, its role in the Spanish setting is yet to be determined. We aimed to estimate the rate and incidence of PIVC failure at Manacor hospital (Spain) as baseline within a wider quality improvement initiative.

**Methods:**

Tips from all PIVC removed during December 2017 and January 2018 in hospital wards were cultured semiquantitatively. The study population included all PIVCs inserted in adult patients admitted to any of three medical and one surgical wards, emergency department, critical care unit and operating rooms. Clinical, microbiological and ward information was collected by clinical researchers for each PIVC from insertion to removal on the study sites. CRBSI was defined per international guidelines (i.e., Centers for Disease Control and Prevention, USA). Data was analysed descriptively.

**Results:**

Seven hundred and eleven tips were cultured, with 41.8% (297/711) reported as PIVC failure. The PIVC failure rate density-adjusted incidence for hospital length of stay (HLOS) was 226.2 PIVC failure/1000 HLOS. 5.8% (41/711) tips yielded positive isolates, with most frequent microorganisms *Staphylococcus spp* (*S. epidermidis* 29/41, 70.7%, *S. aureus* 2/41, 4.9%, *S. hominis* 2/41, 4.9%), and *Acinetobacter baumannii* (1/41, 2.4%). One *S. aureus* isolate was methicillin-resistant. 53.6% (22/41) positive cultures were obtained from patients with local signs and symptoms compatible with catheter-related infection (CRI), 2.4% (1/41) were compatible with CRBSI type 2 and that clinical signs improve within 48 h of catheter removal (density-adjusted incidence for hospital stays of 16.7 PIVC-CRI/1000 hospital-stays and 0.76 PVC-BSI/1000 hospital-stays respectively) and no patients were diagnosed CRBSI type 3 with a bacterial growth concordant in tip and blood cultures. Most cases responded favourably to catheter removal and management.

**Conclusions:**

Our findings show that almost 42% PIVCs resulted in unplanned removal, amplifying the importance in terms of morbidity, mortality and patient safety. A high number of positive tip cultures without clinical signs and symptoms was observed. We underpin the importance to remove unnecessary PIVCs for the prevention of CRBSI.

## Background

Peripheral intravenous catheters (PIVCs) are the most widely used invasive device in hospitals worldwide [1]. These devices can lead patients to experience multiple complications during the insertion, maintenance and management of intravenous therapy during a hospital admission [1–3]. PIVCs are indicated for short-term use, usually around a week. However, up to 69% of PIVCs are prematurely removed due to PIVC failure, defined as unplanned PIVC removal with mechanical complications (phlebitis, occlusion, infiltration) or infection before the completion of any scheduled intravenous therapy [4, 5].

Catheter-related bloodstream infections (CRBSI), which include those associated with the use of central venous catheters (CVCs) and PIVCs, are serious yet preventable adverse events for patients, with a high cost in terms of morbidity and mortality [6–8]. CRBSIs account for ~ 40% of all bloodstream infections (BSI) [3]. Specifically, the incidence of PIVC-BSI is 0.1% or 0.5 per 1000 catheter-days [9]. CRBSIs can prolong HLOS and result in up to 25% attributable mortality rate [10, 11]. Treating each episode of PIVC-BSI costs approximately US$45000 in The excess hospitalization costs associated to the treatment of each episode of PIVC-BSI amount to ~US$45000 [4, 6]. Health organizations have implemented strategies aimed at preventing and reducing PIVC failure rates due to the complications reported [12].

To date, there have been few systematic attempts to measure PIVC failure and CRBSI in hospitals of the Spanish National Health System. Therefore, the main purpose of this study was to estimate the rate and incidence of mechanical and infectious PIVC failure at Hospital Manacor (Spain) as baseline to inform the components of a wider quality improvement initiative in PIVC care due to be implemented in our setting [13]. The secondary objectives were to characterise PIVC failure, describe pathogenic microorganisms isolated from catheter tips, and explore factors associated with the reason for catheter removal and positive culture of isolates.

## Methods

### Study design and participants

We conducted a prospective, observational study at Hospital Manacor which serves a population of 150,000 inhabitants in the eastern sector of Mallorca in Spain. The hospital has 240 beds for all clinical specialities except cardiac, thoracic and neuro surgery. Routine clinical practice in our setting to manage failed catheters does not include recommendations to systematically culture the tip of every PIVC removed. However, in order to determine with accuracy the frequency of catheters failing due to infectious reasons it would be necessary to carry out such serial culturing. Therefore, during the study the tips from all PIVC removed in hospital wards between November 28, 2017 and January 12, 2018 were cultured via the semiquantitative roll plate method, cutting 1.7 to 2.3 cm off the distal segment of PIVC with sterile scissors by clinicians. All tips were sent immediately to the laboratory where tips were rolled 3–4 times onto a blood agar plate at 37 °C for 72 h for culture. The plates were examined daily with bacterial colonies counted as soon as growth was detected. Positive cultures were defined as those with ≥15 colony forming units (CFU). Diagnoses of CRBSI type 2 and type 3 were confirmed if a positive PIVC tip culture was associated with a positive peripheral blood culture for the same microorganism immediately before or within 48 h after catheter removal or a differential time to positivity of blood culture ≥2 h and absence of other infectious focus explaining the positive blood culture result [14]. The local microbiologist followed up each tip culture for 7 days, observing clinical signs and other relevant cultures.

The convenience sample included all PIVCs inserted in adult patients (18-years or older) admitted to any of three medical and one surgical wards, emergency department, and the critical care unit and operating rooms. Information about clinical and microbiological variables together with ward details were collected by the clinical researchers for each PIVC from insertion to removal on each study site. Clinical staff in all wards were notified about the study via face-to-face meetings together with an informative video to facilitate study adoption and implementation. The study was approved by the research ethic committee of Hospital Manacor and Balearic Islands (IB3492/17PI).

### PIVC care and maintenance

All PIVCs were inserted by nurses following current hospital policy. The skin was prepared with 2% chlorhexidine in 70% isopropyl alcohol. All PIVs were non-winged catheters, with a needle-free valve directly connected to 10 cm of extension tubing ending in a three-way connector. A transparent dressing with polyurethane borders was applied at the insertion site to secure the PIVC in situ. All PIVCs were flushed with sterile 0.9% sodium chloride after every use and not used a “scrup-the-hub” technique. Standard caps on all needleless connectors were in place to minimize accidental tubing disconnections. The current policy did not include routine disinfection of PIVC caps as a preventive measure.

### Outcomes

The primary outcome of the study was all-cause PIVC failure, defined previously as unplanned PIVC removal before the completion of therapy. PIVC failure could result from the following adverse events associated with their use: Catheter-related infection (CRI) or CRBSI type 1 (positive culture in tips removed from patients with local signs or symptoms compatible with catheter inserted site infection, type 2 and type 3 (primary BSI without and with laboratory confirmed local PIVC infection respectively, with clinical signs improve within 48 h of catheter removal, defined as per Clinical Practice Guideline of Centers for Disease Control and Prevention, USA [15]), dislodgement (entire PIVC dislodged from the patient’s body), extravasation (inadvertent leakage of a vesicant solution into surrounding tissue), obstruction (complete PIVC occlusion, whereby neither aspiration nor infusion are possible) and phlebitis (defined by at least one or more of the following: persistent pain referred to PIVC, erythema, swelling, palpable thrombosis of the cannulated vein).

Secondary outcomes were subtypes of PIVC failure (CRBSI, dislodgement, extravasation, obstruction and phlebitis), PIVC/hospital length of stay (HLOS) per patient (total number PIVCs during HLOS per patient), PIVC characteristics (insertion site, insertion side, cannula size, insertion ward, dressing and removal setting), indwelling time (time from insertion to removal), PIVC bacterial infection (> 15 colony forming units, CFU), and microorganisms isolated.

### Statistical analysis

The statistical analysis included a description of the sample (continuous data represented by means and standard deviation, and categorical data represented by frequency and percentage tables), and bivariate analysis with parametric and non-parametric tests, depending on the nature of the distributions (correlation, ANOVA, chi-square). For the calculation of incidence density referring to total hospital stay we omitted the HLOS of wards not including during the study period. Data were analysed using SPSS IBM Statistics version 25.

## Results

### Clinical characteristics and outcomes of the sample

During the study period (46 days) we analysed 711 PIVCs from 504 patients, mainly from medical wards (398, 79%). There were 236 (46.8%) female patients with a mean age of 68.5 years (SD, 18.2 years). The median PIVCs per HLOS for the sample was 1.5 PIVCs per patient (SD, 0.9). The incidence density for total hospital admissions was 1313. Regarding total PIVC/HLOS, 504 patients had 1 PIVC (70.9%), 138 carried 2 PIVCs (19.4%) and 69 carried 3 or more PIVCs (9.7%).

**Fig. 1:**
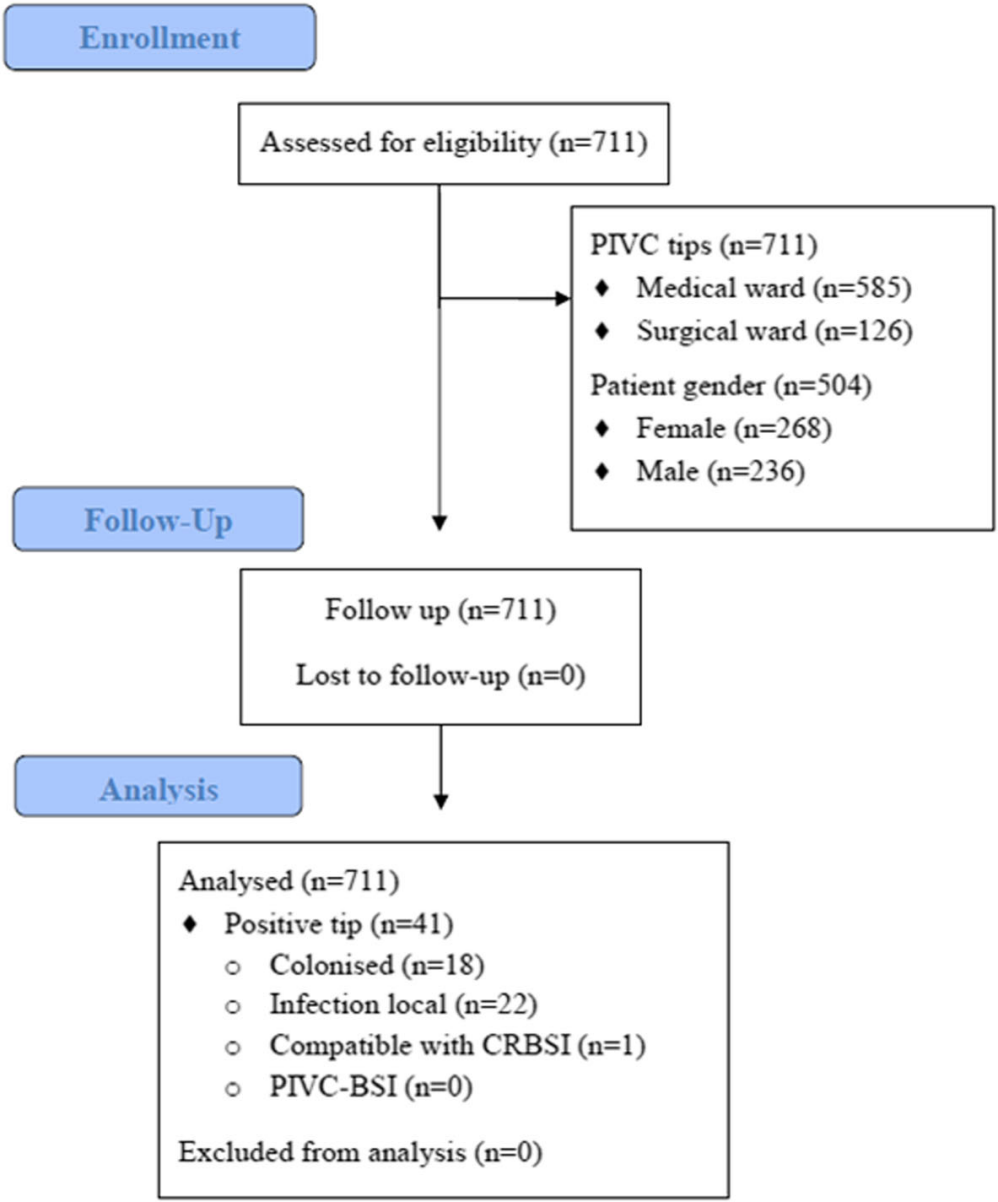
Flow chart. PIVC Failure

We cultured 711 PIVC tips, mostly from patients in medical wards (585, 82.3%). 297 PIVCs (41.8%) were defined as PIVC failure, resulting in a density-adjusted incidence for HLOS of 226.2 PIVC failure/1000 HLOS. 71 PIVCs failures out of 126 PIVC insertions occurred on surgical wards (56.3%), therefore the setting with a highest rate of PIVCs failure. Regarding subtypes of PIVC failure, extravasation and phlebitis accounted for 33.3% of all reasons for removal, and 41/711 (5.8%) positive. 22/41 (53.6%, 16.7 PIVC-CRI/1000 hospital-stays) of those positive cultures were obtained from patients with local signs and symptoms compatible with CRI. Additionally, 1/41 (2.4%, 0.76 PIVC-BSI/1000 hospital-stays) cultures was compatible with CRBSI, with clinical signs improving within 48 h of catheter removal. There were no patients diagnosed with CRBSI with concordant bacterial growth isolated in catheter tip and blood culture (Fig. 1). No serious adverse events were documented during the study.

The isolated microorganisms were *Staphylococcus spp* –*S. epidermidis* 29/41 (70.7%), *S. aureus* 1/41 (2.9%), methicillin-resistant *S. aureus* (MRSA) 1/41 (2.9%), S. hominis 2/41 (4.9%), *S. haemolyticu*s 2/41 (4.9%), *S. capitis* 1/41 (2.9%), *S. simulans* 1/41 (2.9%)–, *Corynebacterium spp*. 1/41 (2.9%), *Candida albicans* 1/41 (2.9%), non-fermenting gram-negative bacillus 1/41 (2.9%) and *Acinetobacter baumannii* 1/41 (2.4%).

There were statistically significant differences between the removal settings regarding female gender (*p* = 0.020), mean PIVC per HLOS (*p* = 0.030), total PIVC per HLOS (*p* = 0.017), PIVC failure (*p* < 0.001) and subtypes of PIVC failure (obstruction, phlebitis, dislodgement, extravasation and suspected infection; p < 0.001). All variables associated with clinical characteristics and outcomes are described in Table 1.

**Table 1:** Clinical characteristics and outcomes of the sample

Demographic characteristics	Medical	Surgical	Overall	*p* value
Total patients, n (%)	398 (79.0)	106 (21.0)	504 (100)	0.297
Patient gender, n (%)				
Female	201 (50.5)	67 (63.2)	268 (53.2)	0.020
Male	197 (49.5)	39 (36.8)	236 (46.8)	
Age years, mean (SD)	68.6 (18.7)	68.0 (16.4)	68.5 (18.2)	0.833
Total PIVC/ HLOS, n (%)				
1	398 (68.7)	106 (80.3)	504 (70.9)	0.017
2	120 (20.7)	18 (13.6)	138 (19.4)	
3	37 (6.4)	5 (3.8)	42 (5.9)	
4	13 (2.2)	2 (1.5)	15 (2.1)	
5	7 (1.2)	1 (0.8)	8 (1.1)	
6	3 (0.5)	0	3 (0.4)	
7	1 (0.2)	0	1 (0.1)	
Total PVCs, n (%)	585 (82.3)	126 (17.7)	711 (100)	
Dressings				
Transparent bordered polyurethane at insertion site	585 (82.3)	126 (17.7)	711 (100)	
Primary outcome				
PIVC failure, n (%)	226/585 (38.6)	71/126 (56.3)	297/711 (41.8)	< 0.001
PIVC failure n per 1000 HLOS	172.1	54.1	226.2	
Secondary outcomes				
Subtype PIVC failure				< 0.001
Obstruction, n (%)	29 (5.0)	15 (11.9)	44 (6.2)	
Obstruction n per 1000 HLOS	22.1	11.4	33.5	
Phlebitis	79 (13.4)	28 (21.4)	107 (15.0)	
Phlebitis n per 1000 HLOS	60.2	21.3	81.5	
Dislodgement	8 (1.4)	3 (2.4)	11 (1.5)	
Dislodgement n per 1000 HLOS	6.1	2.3	8.4	
Extravasation	107 (18.3)	23 (18.3)	130 (18.3)	
Extravasation n per 1000 HLOS	81.5	17.5	99.0	
Suspected infection	3 (0.5)	2 (1.6)	5 (0.7)	
Suspected infection n per 1000 HLOS	2.3	1.5	3.8	
Negative tip	553 (94.5)	117 (92.9)	670 (94.2)	0.683
CRBSI (> 15 CFU), n (%)	32 (5.5)	9 (7.1)	41 (5.8)	
CRBSI n per 1000 HLOS	24.4	6.8	31.2	
Colonised	13 (40.6)	5 (55.6)	18 (43.9)	
Colonised n per 1000 HLOS	9.9	3.8	13.7	
CRBSI 1	18 (56.3)	4 (44.4)	22 (53.7)	
CRBSI 1 n per 1000 HLOS	13.7	3.0	16.7	
CRBSI 2	1 (3.1)	0	1 (2.4)	
CRBSI 2 n per 1000 HLOS	0.7	0	0.7	
CRBSI 3	0	0	0	
CRBSI 3 n per 1000 HLOS	0	0	0	
Isolated microorganisms, n (%)	32 (100)	9 (100)	41 (100)	0.153
*Staphylococcus aureus*	1 (3.1)	0	1 (2.4)	
*MRSA*	1 (3.1)	0	1 (2.4)	
*Staphylococcus epidermidis*	23 (71.9)	6 (66.7)	29 (70.7)	
*Staphylococcus haemolyticus*	0	2 (22.2)	2 (4.9)	
*Acinetobacter baumannii*	0	1 (11.1)	1 (2.4)	
*Staphylococcus hominis*	2 (6.3)	0	2 (4.9)	
*Staphylococcus capitis*	1 (3.1)	0	1 (2.4)	
*Candida albicans*	1 (3.1)	0	1 (2.4)	
*Corynebacterium sp.*	1 (3.1)	0	1 (2.4)	
*Staphylococcus simulans*	1 (3.1)	0	1 (2.4)	
Non-fermenting gram-negative bacillus	1 (3.1)	0	1 (2.4)	

Table 2 offers information about the reasons for catheter failure. Overall, 297/711 (41.8%) PIVCs resulted in catheter failure. Patients over 65 years (202/297; 68.0%) presented high rate of PIVC unplanned removal and comorbidity was observed. In addition, they had 2 or more comorbidities (451/711; 63.4%). 335/711 (47.1%) of PIVCs were inserted into an area of non-flexion, such as the forearm. The majority of PIVCs (465/711, 65.4%) were intravenous cannula size 20G and all 711 PIVCs were secured with transparent polyurethane dressing. A high number of PIVCs (329/711, 46.3%) had an indwelling time ranging 48–96 h before removal. PIVCs were inserted primarily at the emergency department (333/711, 46.8%) or at hospital wards (263/711, 37%). 55 (7.8%) PIVC insertion ward data was not registered due to fragmentation of electronic records between emergency and hospital wards. The variables related to age group (*p* = 0.456), comorbidity (*p* = 0.686) location (*p* = 0.210), laterality (*p* = 0.472), intravenous cannula size (*p* = 0.452), indwelling time (*p* = 0.599) and clinical area of insertion (*p* = 0.230) were not statistically significant in relation to PIVC failure. However, a statistically significant association was observed between catheter failure and setting of removal (p = < 0.001).

**Table 2:** Comparative analysis of characteristics by PIVC Failure

Variables	PIVC Failure			p
	No	Yes	Overall	
Total PVCs, n (%)	414 (58.2)	297 (41.8)	711 (100)	
Age years, n (%)				
18–34	26 (6.3)	16 (5.4)	42 (5.9)	0.456
35–49	29 (7.0)	31 (10.4)	60 (8.4)	
50–64	76 (18.4)	48 (16.2)	124 (17.4)	
65–79	134 (32.3)	89 (30.0)	223 (31.4)	
+ 80	149 (36.0)	113 (38.0)	262 (36.8)	
Comorbidity, n (%)				
0	50 (12.1)	38 (12.8)	88 (12.4)	0.686
1	105 (25.3)	67 (22.6)	172 (24.2)	
2 or more	259 (62.6)	192 (64.6)	451 (63.4)	
Insertion site, n (%)				
Hand	109 (26.3)	64 (21.5)	173 (24.4)	0.210
Wrist	14 (3.4)	8 (2.7)	22 (3.1)	
Forearm	200 (48.3)	135 (45.5)	335 (47.1)	
Antecubital fossa	89 (21.5)	88 (29.6)	177 (24.9)	
Arm	1 (0.2)	0	1 (0.1)	
Foot	1 (0.2)	2 (0.7)	3 (0.4)	
Laterality, n (%)				
Left	219 (52.9)	149 (50.2)	368 (51.8)	0.472
Right	195 (47.1)	148 (49.8)	343 (48.2)	
Intravenous cannula size, n (%)				
16 G	3 (0.7)	2 (0.7)	5 (0.7)	0.452
18 G	87 (21.0)	58 (19.5)	145 (20.4)	
20 G	275 (66.4)	190 (64.0)	465 (65.4)	
22 G	46 (11.1)	41 (13.8)	87 (12.2)	
24 G	3 (0.7)	6 (2.0)	9 (1.3)	
Indwelling, n (%)				
< 48 h	130 (31.4)	104 (35.0)	234 (32.9)	0.599
48–96 h	196 (47.3)	133 (44.8)	329 (46.3)	
> 96 h	88 (21.3)	60 (20.2)	148 (20.8)	
Setting of Insertion, n (%)				
Ward hospital	153 (37.0)	110 (37.0)	263 (37.0)	0.230
Operating room	32 (7.7)	14 (4.7)	46 (6.5)	
Emergency	186 (44.9)	147 (49.5)	333 (46.8)	
ICU	3 (0.7)	2 (0.7)	5 (0.7)	
Maternity	4 (1.0)	2 (0.7)	6 (0.8)	
Other hospitals	1 (0.2)	0	1 (0.1)	
Primary care	2 (0.5)	0	2 (0.3)	
Not registered	33 (8.0)	22 (7.4)	55 (7.8)	
Setting of removal				
Medical wards	359 (86.7)	266 (76.1)	585 (82.3)	< 0.001
Surgical wards	55 (13.3)	71 (23.9)	126 (17.7)	
Negative tip	398 (96.1)	272 (91.6)	670 (94.2)	0.010
CRBSI (> 15 CFU), n (%)	16 (3.9)	25 (8.4)	41 (5.8)	
Colonised	16 (100)	2 (8.0)	18 (43.9)	< 0.001
CRBSI type 1	0	22 (88.0)	22 (53.6)	
CRBSI type 2	0	1 (4.0)	1 (2.4)	
CRBSI type 3	0	0	0	
Isolated microorganisms, n (%)	16 (100)	25 (100)	41 (100)	0.822
*Staphylococcus aureus*	1 (6.2)	0	1 (2.4)	
*MRSA*	0	1 (4.0)	1 (2.4)	
*Staphylococcus epidermidis*	12 (75.0)	17 (68.0)	29 (70.7)	
*Staphylococcus haemolyticus*	1 (6.2)	1 (4.0)	2 (4.9)	
*Acinetobacter baumannii*	0	1 (4.0)	1 (2.4)	
*Staphylococcus hominis*	1 (6.2)	1 (4.0)	2 (4.9)	
*Staphylococcus capitis*	0	1 (4.0)	1 (2.4)	
*Candida albicans*	0	1 (4.0)	1 (2.4)	
*Corynebacterium sp.*	1 (6.2)	0	1 (2.4)	
*Staphylococcus simulans*	0	1 (4.0)	1 (2.4)	
Non-fermenting gram-negative bacillus	0	1 (4.0)	1 (2.4)	

Table 3 describes factors associated with the reason for PIVC removal related to positive tip cultures. 18/41 (43.9%) positive tips were associated unnecessary PIVCs, which came from patients discharged or intravenous therapy completed and removed. 23/41 (56.1%) positive tips related to PIVC failure (obstruction, phlebitis and extravasation). A higher number of positive tips (16/41, 39.1%) identified as obstruction with insertion site inflammation and extravasation compared to phlebitis rates were observed.

**Table 3:** Diagnosis of positive tip cultures relating the removal reasons

	CRBSI			
	colonised	type 1	type 2	type 3
Intravenous therapy completed, or patient discharged, n (%)	18 (43.9)			
Obstruction with insertion site inflammation, n (%)		4 (9.8)	0	0
Phlebitis, n (%)		6 (14.6)	1 (2.4)	0
Extravasation, n (%)		12 (29.3)	0	0
Suspected infection, n (%)		0	0	0

## Discussion

In this study almost 42% PIVCs resulted in failure, a rate comparable to previous studies reporting 28–55% [16–20]. Few studies have dealt however with PIVC failure following up CRBSI with microbiological culture of catheter tips in medical and surgical hospital settings. This approach allowed us to estimate with accuracy and reliability the rate and incidence of PIVC failure at Hospital Manacor, including the variety of subtypes of PIVC failure, and pathogenic microorganisms isolated on catheter tips. We observed higher PIVC failure, phlebitis and obstruction rates in surgical wards, which may be explained by the administration of intravenous therapy in surgical patients, usually a higher volume of antibiotics and analgesia in a short period of time [21–23]. However, the female gender was associated with a higher PIVC failure rate in the surgical setting [22]. Therefore, we cannot directly attribute the cause to the setting alone omitting the gender variable.

PIVC insertion care, maintenance and management of intravenous therapy are common care interventions. Therefore, PIVC failure disrupts intravenous therapy workflow requiring a new PIVC placement, a situation with potential for multiple complications, pain and distress for patients plus an important demand on health system resources [21, 22, 24]. To match the vascular access devices (VAD) to the therapy prescribed and reduce the devices unnecessarily inserted nurses must take into account patient characteristics and type and duration of treatment. In our study, 39% of patients carried more than two PIVCs during their admission. The inadequacy of the device suggests a negative patient experience and poor-quality care. The availability of an algorithm to optimally select a VAD could contribute towards avoidance of PIVC failure and the deterioration of vessel health. The PIVC reinsertion impose a considerable consumption of clinical resources and time. For example, and focusing just on staff costs, if the average annual salary of a registered nurse is currently US$73,550 (or ~US$35.30 per hour), and estimating the time employed for each PIVC reinsertion as approximately ten minutes, then each PIVC failure would result in ~US$13,336 per year. However, the majority of costs are indirect and relate to therapy and increased HLOS associated to CRBSIs [25, 26].

Our study reported a low occurrence of CRBSI (2.4%), and no PIVC-attributable BSI. These results are encouraging, in comparison with previous studies which obtained one CRBSI in 6538 PIVCs [27] and one episode of CRBSI in 5907 PIVCs [28]. However, a significant number of positive tip cultures were obtained at removal following completed intravenous therapy or at patient discharge. It is remarkable that we did not observe a greater CRBSI rates after obtaining high colonization rates. This may be due to the safety culture implemented for the early removal of PIVCs when intravenous therapy is completed, as well as cases of unnecessary insertions, by the infection control team. Despite the low incidence of CRBSI type 2 detected (1/711, 0.7 per 1000 HLOS) in our study, the volume PIVCs used warrants continued attention and skilled care for its potential in terms of morbidity, mortality and patient safety [29–32].

Traditionally, PIVCs had been given limited relevance within CRBSI prevention strategies, underestimating the magnitude of the problem [33]. CRBSIs have been more frequently associated with medium and long-term central intravenous devices [34, 35]. Theoretically, catheter indwelling time is one of the major risk factors for PIVC failure [21]. However, a Cochrane review concluded that routine removal of indwelling PIVC did not reduce the risk of CRBSI [36]. and therefore eliminated the recommendation of routine removal of catheters at 72-96 h, leaving the decision to the clinical judgment of nurses [28]. Catheter removal is recommended when clinical manifestations of catheter failure are detected. However, we do not always eliminate the cause of infection when we detect general symptoms related to CRBSI, removing PIVC immediately. We observed that there were no statically significant differences in indwelling time in our analysis of characteristics of PIVC failure. Previous studies confirmed that it is the overall exposure of the PIVC use that increases risk [28]. In our study, all PIVCs were appropriately dressed with transparent bordered polyurethane at the insertion site to anchor the catheter. When PIVCs are not properly secured, micromotions may encourage migration of microorganisms along the catheter, leading to CRBSI [21, 37]. The low incidence of CRBSI (2.4%) and the absence of PIVC-attributable BSI in our study, despite the presence of many PIVCs isolated with pathogen microorganisms, do not support systematically performing tip cultures of PIVCs for the prediction of CRBSI. The therapeutic approach to local clinical signs of PIVC failure, such as extravasations, insertion site inflammations and phlebitis, should be the removal of PIVCs with the observation of the onset of systemic symptoms. Furthermore, we recommend the premature remove of unnecessary PIVC when intravenous therapy had been completed, as such an effective therapeutic approach for prevention CRBSI.

Our study presents some limitations that must be taken into account before interpreting the results. Firstly, we conducted a two-month prospective observational study in a single facility, being a study with a relatively short period of time due to the budget to carry out serial catheter tip cultures. Future research must consider to the implementation of a multimodal intervention will decrease the incidence of PIVC failure associated in adult inpatients, analysing the fidelity to the recommendations within clinical practice guidelines for insertion and management of PIVCs.

## Conclusions

Our findings indicate that almost half of the PIVCs required unplanned removal. Although potentially fatal adverse events such as CRBSIs have a low incidence in our study, it is remarkable in terms of morbidity, mortality, patient safety and additional clinical workload, mainly for nurses. From the point of view of quality of care, we recommend that organizations emphasize increasing the improvement initiatives within a wider total quality process that includes adequacy of vascular access device, optimal PIVC insertion care, maintenance and management of intravenous therapy, and proactive pursuit of premature removal opportunities, underpinning the importance of removing unnecessary PIVCs.

## Data Availability

The datasets analysed during the current study are available from the corresponding author on a request from group investigation.

## Abbreviations

BSI: Bloodstream infection
CFU: Colony forming units
CRBSI: Catheter-related bloodstream infection
CRI: Catheter-related infection
CVC: Central venous catheter
HLOS: Hospital length of stay
MRSA: Methicillin resistant *Staphylococcus aureus*
PIVC: Peripheral intravenous catheter
VAD: Vascular access device
